# Trauma and orthopaedic surgery response at the world’s largest sporting event: an analysis of the FIFA World Cup Qatar 2022

**DOI:** 10.1007/s00264-023-05843-5

**Published:** 2023-05-30

**Authors:** Loay A. Salman, Osama Z. Alzobi, Ashraf T. Hatnouly, Yaman Al Haneedi, Maamoun Abousamhadaneh, Shamsi Hameed, Mohamed Al Ateeq Al Dosari, Ghalib Ahmed

**Affiliations:** 1 Orthopedics Department, Surgical Specialty Center, Hamad General Hospital, Hamad Medical Corporation, PO Box 3050, Doha, Qatar; 2 College of Medicine, Qatar University, Doha, Qatar

**Keywords:** Orthopaedics, Training, FIFA, World Cup, Sports, Qatar

## Abstract

**Purpose:**

The purpose of this study is to analyse the impact of the FIFA World Cup Qatar 2022 on the Orthopaedic Surgery department at Hamad Medical Corporation and its response to the challenges posed by the world’s largest sporting event.

**Methods:**

A retrospective analysis was conducted on the epidemiology, crisis management plan, and training program adaptations at the Orthopaedic Surgery department during the World Cup. Descriptive analysis of the number and types of surgeries performed, patient demographics, and the disaster preparedness plan were performed.

**Results:**

During the tournament period (November–December 2022), 706 patients (4.22% football fans) were operated on, with an average age of 44 ± 17 years. Most patients were males, 67%. Of the 706 patients, 60.33% were emergency cases, 38.24% were elective, 1.27% were limb-saving, and one life-saving procedure was performed, comparable to pre-tournament numbers. The patients were of 77 different nationalities, reflecting the diverse background of Qatar’s population and the international fanbase of the tournament.

**Conclusions:**

This analysis provides valuable insights for future mega sporting events and highlights the importance of crisis management and training program adaptation for optimal patient care and resident training advancement. The findings demonstrate the crucial role of the Orthopaedic Surgery Department in responding to the challenges posed by large-scale events.

## Introduction

The FIFA World Cup Qatar 2022 was the largest sporting event ever organized in the Middle East. The influx of over 1.4 million visitors to the state of Qatar, equivalent to 52% of the country’s entire population, placed significant pressure on all sectors, including the healthcare system [1]. Therefore, Qatar invested in healthcare infrastructure to enhance its capacity and preparedness in preparation for the World Cup [2, 3].

Although hosting such events provides tremendous economic and touristic opportunities [3], it can also strain healthcare systems, particularly post-COVID-19 [4], and raise concerns about safety due to potential incidents of aggressive fan behaviour [5].

At Hamad Medical Corporation, the only level-one trauma centre in the country, the Orthopaedics Surgery Department established a special task force and adopted a crisis plan during the tournament period. The aim was to provide optimal patient care, ensure the well-being of staff and trainees, and maintain the smooth running of the Orthopaedic residency and fellowship training programs in accordance with the Accreditation Council for Graduate Medical Education-International (ACGME-I) core competencies.

This article highlights epidemiological insights, discusses the departmental strategy plan, and analyses the event’s impact on resident training progress. In addition, it stresses the lessons learned during the World Cup period to facilitate a well-coordinated response to similar events in the future.

## Methods

### Data source

Data from HMC theatre electronic case logs was retrieved retrospectively from January 1, 2022, to December 31, 2022, for internal review and analysis. The search and data review was conducted independently by two authors. The collected data included the patient ID number, age, gender, nationality, injury type, injury mechanism, local versus visitor status, surgery date, surgery type (emergency, elective, limb-saving, and life-saving), operative time, admission, operation, and discharge dates.

### Statistical analysis

Data analysis was conducted using IBM SPSS version 24 (SPSS Inc., Chicago, IL, USA). Measures of central tendency, including the mean, range, and percentage, were used to summarize the patients’ demographics. *t*-test was utilized for continuous variables, and a *p*-value of less than 0.05 was considered significant. No power analysis was performed as all patients who underwent orthopaedic surgery during November to December 2022 were included in the study.

## Results

### Epidemiological insights

A total of 706 patients were included in this analysis, with a mean age of 44 ± 17 years. Most patients were males (67%), and 33% were females. Around 96% of the patients were residents of Qatar, whereas visitors represented a mere 4% of the cases. Out of the 706 patients, 426 (60.33%) were emergency cases (fractures and soft tissue injuries), while 270 (38.24%) were elective cases. Nine (1.27%) limb-saving and one life-saving procedure were performed among these cases. These figures were comparable to the pre-tournament numbers, with an average of 345 ± 41 operated cases per month in the rest of 2022 (*t*-test = 0.2645, *df* = 10, *p*-value = 0.7967). The mean operative time was 1.8 ± 1.29, and the average length of hospital stay was 6.2 ± 8.84. Of note, 77 different nationalities were reported, reflecting the diverse background of Qatar’s population and the international fans attending the World Cup tournament. A summary of patients’ demographics is shown in Table 1.

**Table 1:** A summary of baseline patients’ demographics

Patient’s characteristics	Frequency	Percentage
Overall no. patients	706	100.00%
Mean age (years)	44 ± 17	
Gender		
Male	473	67.00%
Females	233	33.00%
Emergency cases	426	60.33%
Elective cases	270	38.24%
Limb-saving procedures	9	1.27%
Life-saving procedures	1	0.14%
No. fractures	275	39.00%
Soft tissue injury	71	10.00%
Amputation	4	
Below knee	2	
Above Knee	2	
Operative time (hours)		
Mean(± SD)	1.8 ± 1.29	
Length of stay (days)		
Mean (± SD)	6.2 ± 8.84	
No. nationalities	77	
Residency status		
Residents	676	96.00%
Visitors	30	4.00%

**Fig. 1:**
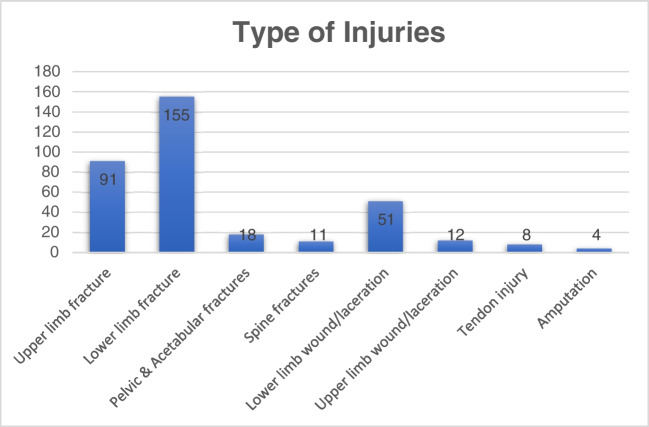
A summary of the type of injuries among patients that required emergency orthopaedic procedures

Furthermore, the most frequent injury pattern was lower limb fractures (155), followed by upper limb fractures (91), lower limb wound lacerations (51), pelvic and acetabular fractures (18), spine fractures (11), and other soft tissue injuries. A detailed breakdown of the types of injuries encountered is presented in Table 2 and Fig. 1.

**Table 2:** A summary of injury types that required surgery on an emergency basis

Type of injury	Frequency
Upper limb fracture	91
Humerus (proximal, shaft)	17
Elbow fractures	15
Radius & ulna shaft	20
Distal radius	31
Scaphoid	3
Clavicle	5
Lower limb fracture	155
Hip	12
Femur	43
Tibia fractures	39
Ankle fractures	31
Patella	4
Calcaneus fractures	11
Talus fracture	2
Forefoot fractures	13
Pelvic and acetabular fractures	18
Spine fractures	11
Lower limb wound/laceration	51
Upper limb wound/laceration	12
Tendon injury	8
Amputation	4
Below knee	2
Above knee	2

ASPETAR, a FIFA-affiliated centre, was designated to provide medical care for professional athletes and footballers during the World Cup. Of the 32 participating national teams, 28 reported player injuries, affecting a total of 72 (8.64%) players, assuming each team had 26 players. Knee injuries were the most frequent type, accounting for 30.56% of the total, followed by thigh injuries (19.44%), ankle and foot injuries (15.28%), calf and leg injuries (15.28%), and eight other types of injuries (11%). The national teams with the highest number of injuries were Saudi Arabia (9.72% of the total, with 7 cases), France (8.33% of the total, with 6 cases), and Portugal and Morocco (6.94% of the total, with 5 cases each) [6]. A detailed breakdown of the types of injuries is presented in Table 3.

**Table 3:** A breakdown of various injuries suffered by football players during the 2022 FIFA World Cup, as reported by national teams

Injury pattern	No of cases	Percentage
Knee injury	22	30.56%
Thigh injury	14	19.44%
Foot and ankle	11	15.28%
Leg and calf	11	15.28%
Shoulder injury	2	2.78%
Groin and pelvic	2	2.78%
Hand and wrist	1	1.39%
Head and face	1	1.39%
Others	8	11.11%

## Discussion

### Crisis management plan and preparation

Effective crisis management is crucial to ensure the safety of athletes, visitors, and spectators during major sports events. To address this, the Orthopaedic Department and Bone and Joint Center of Hamad Medical Corporation (HMC) established a disaster preparedness plan for the World Cup 2022. The Orthopaedic Disaster Preparedness Team (ODPT) was formed as part of this plan. The ODPT consisted of leaders from various disciplines and subspecialties responsible for managing and executing a response to any major incidents that may arise.

The ODPT was divided into four primary teams: Emergency Department (ED), Operating Theatre (OT), Inpatient, and Outpatient teams. Each team comprised leading consultants, specialists, fellows, and residents. The ED team managed orthopaedic trauma cases at the emergency department and ensured efficient patient care. Additionally, the team evaluated ED patients, handled admissions and discharges, made referrals to other facilities, coordinated with the disaster committee lead, and worked with other teams.

The OT team ensured the availability of personnel, supplies, operating rooms, and equipment for orthopaedic surgical procedures. The inpatient team evaluated patients; ensured adequate personnel, beds, supplies, and equipment in orthopaedic wards; provided follow-up instructions; and coordinated transfers with other facilities. The outpatient team provided necessary supplies and equipment for ambulatory procedures and outpatient follow-ups in clinics. To facilitate effective communication during any major incident, the leaders of each team established primary and backup communication pathways via designated landlines, bleeps, and smartphone applications.

A daily backup roster consisting of a senior consultant, two specialists, two fellows, and three residents was implemented to ensure workforce availability. Furthermore, the ODPT conducted regular disaster drills and simulation exercises before the World Cup to enhance the preparedness and response of residents and other orthopaedic personnel. These exercises aimed to imitate actual event responses as closely as possible and provide simulation-based training activities.

By implementing these measures, the HMC Orthopaedic Department and Bone and Joint Center aimed to provide a smooth response to potential crises during the World Cup 2022. The disaster preparedness plan and the ODPT’s efforts ensured that patients received timely and effective orthopaedic care, thereby ensuring the safety of athletes, visitors, and spectators during the event.

### Training program adaptations

Integrating residency and fellowship programs with major sporting events presents trainees with numerous advantages, particularly for those seeking subspecialty fellowships in sports medicine and orthopaedic trauma, where exposure to such events is essential [7, 8]. The FIFA World Cup Qatar 2022 offers a unique opportunity to enhance residency education through exposure to various clinical and surgical cases.

Some studies have suggested that sports event coverage can be considered essential in Orthopedic residency programs [8, 9]. For instance, a survey by Hodax et al. [9] found that 89% of residency programs either authorized or required residents to participate in sports event coverage. This experience has boosted residents’ confidence in treating orthopaedic conditions in the field.

At Hamad Medical Corporation, the orthopaedic residency program has adapted to the 2022 FIFA World Cup in accordance with the international accreditation council for graduate medical education’s six core competencies, including professionalism, knowledge, patient care, system-based practice, practice-based learning, and communication skills [10]. The program’s leaders ensured the continuity of the educational process and divided the residents into four groups within the ODPT, providing specialized training to each group. Additionally, the program maintained its educational activities, such as Orthopaedics grand rounds, journal clubs, and didactic lectures, throughout the World Cup for both residents and faculty. Trainees continued their orthopaedic subspecialty training without any disruption in their clinical rotations.

The residency program’s exposure to patients from 77 different nationalities with varying cultural backgrounds provided a unique and enriching experience. Dealing with patients from diverse backgrounds increased the volume of cases and challenged the residents to develop their communication skills as they interacted with patients who spoke different languages and had varying cultural beliefs. This exposure allowed the residents to better understand the complexities of providing patient-centred care and improved their ability to work in a culturally diverse environment. Furthermore, the residents could sharpen their surgical skills through the increased volume of cases and the diverse range of conditions they were exposed to. This experience will undoubtedly serve as a valuable asset in their future careers as practising orthopaedic surgeons.

### Insights and perception

The results of the Orthopedic Surgery Department’s response to the World Cup in Qatar align with previous research on the impact of large-scale sporting events on healthcare systems, where significantly increased patient volume and a greater demand for additional resources, including staff and equipment, are often observed [11, 12].

Moreover, our findings support the importance of advanced planning and preparation in the healthcare sector for large-scale sporting events. Establishing a special task force and implementing a crisis plan allowed the Orthopaedic Surgery section to effectively manage the surge in demand for its services during the World Cup. These efforts also ensured the uninterrupted continuation of the residency and fellowship training programs, which are critical for developing the next generation of orthopaedic surgeons.

Finally, the diversity of nationalities among the patients treated underscores the need for culturally competent healthcare services. This is especially significant in global events like the World Cup, where attendees come from diverse countries and cultural backgrounds.

## Conclusion

The Orthopaedic response was closely integrated with the overall medical response for the tournament, with clear communication and coordination between the various teams. This ensured a seamless and efficient flow of patients and the availability of the right resources at the right time. Furthermore, the World Cup response analysis highlights the importance of crisis management and training program adaptations for optimal patient care and resident training advancement and provides valuable insights for the organization of future mega-sporting events.

## Data Availability

Available upon request.
